# Adherence to Follow-Up and the Related Factors of Paediatric Glaucoma at a Tertiary Care Centre in Western Saudi Arabia

**DOI:** 10.7759/cureus.44124

**Published:** 2023-08-25

**Authors:** Rawan Tash, Reema Alshugaig, Heba Mahboob, Lina H Raffa, Hanan Jamjoom, Nawaf Almarzouki, Ahmed Bawazeer

**Affiliations:** 1 Faculty of Medicine, King Abdulaziz University Faculty of Medicine, Jeddah, SAU; 2 Ophthalmology, King Abdulaziz University Hospital, Jeddah, SAU

**Keywords:** compliance, adherence, children, tertiary hospital, intraocular pressure, primary congenital glaucoma

## Abstract

Introduction: Glaucoma is a main cause of blindness globally. In Saudi Arabia, congenital glaucoma is believed to affect 1 in every 2500 live births. In this study, we identified the adherence and evaluated the factors associated with non-compliance (non-adherence) to recommended follow-up appointments at King Abdulaziz University Hospital, a tertiary centre in Jeddah, Saudi Arabia.

Methods: The medical records of paediatric patients diagnosed with glaucoma between 2009 and April 2022 were reviewed retrospectively. Demographic information, visit dates, scheduled follow-ups, and specific patients’ glaucoma histories were all extracted from the records. Patients were categorized as adherent, non-adherent, or lost to tertiary follow-up (LTTF).

Results: Of 91 patients, 52 (57.1%) were adherent, 20 (22%) were non-adherent, and 19 (20.9%) were LTTF. Most adherent patients were Saudi (59.3% adherent, 26.5% non-adherent, 14.2% LTTF) (P = 0.02). Adherent patients were far likelier to live further away from the ophthalmology clinic (P = 0.03). The frequency of clinical encounters was statistically significantly different between adherence statuses. The non-adherent group had a higher average number of prescribed ocular medications (P = 0.03). The adherent patients had more frequent visits with elevated intraocular pressure (P = 0.02).

Conclusion: A significantly high percentage of paediatric glaucoma patients were non-adherent to follow-up visits. When determining the non-compliance risk among paediatric glaucoma patients, physicians must consider the factors contributing to adherence status, which include nationality, distance to the ophthalmology clinic, and number of prescribed ocular medications.

## Introduction

Childhood glaucoma remains a leading cause of blindness, where 13.5% and 4% of patients become blind unilaterally and bilaterally, respectively [1]. Glaucoma affects more than 300,000 children globally and is frequently linked to profound vision loss (two-thirds of affected children are blind) [2]. Congenital glaucoma is a developmental glaucoma that develops before a child is three years old due to an obstruction that limits sufficient aqueous humour drainage that stems from abnormal trabecular meshwork growth and anterior chamber angle [3].

The reported incidence in a series in the United States was 2.29 per 100,000 [4]. In comparison, congenital glaucoma incidence is markedly higher in the Middle East, including Saudi Arabia, which has more frequent consanguineous marriages than Western countries [3]. The projected incidence of congenital glaucoma in Saudi Arabia is 1 per 2500 live births [5]. Children who inherit familial patterns of the disease frequently present with severe illnesses requiring numerous operations and lifetime care [5]. Therefore, recent developments in biochemical and genetic research, the development of novel diagnostic instruments, the introduction of intraocular pressure (IOP) lowering drugs, and improvements in surgical methods have contributed to a better understanding of this blinding condition and the preservation of affected children's vision [3,6].

While quality of life is highly individualized, adherence to glaucoma therapy is linked to improved quality of life [7]. Typically, it is challenging for patients to comply with recommended glaucoma therapies, which significantly contributes to glaucoma-related visual loss [8]. Ophthalmologists are beginning to understand the burden of glaucoma on adult patients and that non-adherence is more common than previously believed [7]. The issue of medication adherence affects many chronic diseases and is influenced by several variables. Patients can experience difficulties obtaining, using, or dosing drugs [9]. Furthermore, patients might not be convinced that their medication is effective, which is a frequent opinion among patients with asymptomatic glaucoma [9]. Patients' motives for adhering to treatment regimens might be increased by a patient-specific strategy to therapy that includes initial instruction about the negative effects of non-adherence and ongoing feedback about drop efficacy [7].

In 2015, the Stanford University Department of Ophthalmology reported on the significance of a frequently disregarded form of compliance: adherence to advised follow-up visits. Patients who complied with therapy but neglected their scheduled follow-up appointments were likelier to deteriorate clinically, with decreased visual acuity, and might even lose their eyesight [10]. A similar paper that involved a retrospective review of 176 patients with paediatric glaucoma [11] reported exceptionally low adherence to follow-up appointments, where 43% of patients were lost to tertiary follow-up and 3% were non-adherent. The study also noted the significantly decreased follow-up in non-white patients and those who lived farther away from the clinic [11]. Paediatric glaucoma patients encounter an ongoing challenge in adhering to therapy, and attending periodic follow-ups can be strenuous, which can result in the manifestation of additional systemic comorbidities in such patients. However, published data evaluating the follow-up percent adherence or the limitations that might hamper follow-up adherence in paediatric glaucoma patients are limited. The present study aimed to identify the percent adherence among King Abdulaziz University Hospital (KAUH) paediatric glaucoma patients and evaluate the factors associated with non-compliance with recommended follow-up appointments.

## Materials and methods

Patients and study design

This retrospective record review study was conducted at KAUH, a tertiary centre in Jeddah, Saudi Arabia. The medical records of 206 paediatric patients who visited KAUH between 2009 and 2022 were retrieved. Of these, the records of 111 patients were reviewed. Ninety-five patients who did not fulfil the inclusion criteria [age < 18 years with a recorded International Classification of Disease (ICD) coding (ICD9 and ICD10) of juvenile glaucoma, congenital glaucoma, uveitic glaucoma, aphakic glaucoma, or glaucoma secondary to other causes] were excluded from the initial sample. Patients were also excluded if they were aged >18 years, had missing data, had suspected glaucoma, or were diagnosed with other ocular diseases such as retinoblastoma.

Data collection

The information retrieved from the medical records included demographic data (age, date of birth, gender, nationality, city and district of residence). The dates of the first visit and last three visits, planned follow-up dates and frequency of visits per month were reviewed. Specific patient details (IOP of both eyes, which eye was presenting with glaucoma, glaucoma type, glaucoma medications, ocular medications, number of visits with IOP > 21 mmHg) were also extracted. Other information (indication for examination under anaesthesia [EUA] and surgery, total number of surgeries, other presenting illnesses, non-ocular medications, vision in both eyes) was noted. The primary outcomes were the assessment of factors influencing follow-up and the measurement of percent adherence to appointments. We calculated the distance from each patient’s home to the clinic using Google Maps and their address. Each patient’s last best-corrected visual acuity (BCVA) was recorded and classified as good, fair, or poor (Table 1). Adherence to the last and second-to-last visits was classified based on adherence to the designated follow-up date for their following ophthalmology appointment established for the last two visits before 11 April, 2022. Adherence was classified as adherent to one visit, non-adherent (NA), or lost to tertiary follow-up (LTTF) if patients followed up within 0-30 days, between 31 and 180 days, later than 180 days after the recommended appointment time, or never, respectively.

**Table 1:** BCVA classification BCVA = best-corrected visual acuity, CSM = central, steady, and maintained fixation; CSuM = central, steady, and unmaintained fixation.

BCVA category	Fixation	Optotype
Good	CSM at age < 6 months	Better than 20/80
Fair	CSM at age > 6 months	20/200 to 20/80
Poor	CSuM or worse	Worse than 20/200

Ethical approval

The study followed the tenets of the 1975 Declaration of Helsinki that was revised in 2013 and was approved by the KAUH Research Ethics Committee (Ref: 551-21). The requirement for informed consent was waived given the retrospective nature of the study.

Statistical analysis

The Statistical Package for the Social Sciences (SPSS) 26 (IBM, Armonk, NY, USA) was used for data entry and statistical analysis. Continuous variables were described with means and standard deviations, while numbers and percentages were used for categorical variables. A two-tailed P < 0.05 was considered statistically significant. The strength of the relationship between adherence status and patient factors (nationality, distance to the clinic, last BCVA) was measured with a multiple logistic regression model. Odds ratios were estimated with 95% confidence intervals. P < 0.05 was considered significant.

## Results

Patient characteristics

A total of 111 patients were identified following the hospital record review. Of these, 20 were excluded from the analysis due to the lack of glaucoma diagnosis or insufficient data. The remaining 91 patients had paediatric glaucoma (49 boys [54%] and 42 girls [46%]).

Outcome measure

Of the 91 paediatric glaucoma patients, 52 (57.1%) were adherent, 20 (22%) were NA, and 19 (20.9%) were LTTF. Tables 2, 3 present the patients’ clinical and sociodemographic characteristics. Table 4 presents the potential risk factors for nonadherence based on the three adherence groups, respectively. Adherent patients were more likely to be of Saudi nationality (adherent, 73%; NA, 85%; LTTF, 47%, P = 0.02). The groups had similar gender and age distributions, where there was no statistically significant difference in the association with adherence status. Adherent patients were far more likely to live farther away from the ophthalmology clinic (40.16 km away vs. 23.31 km [NA] vs. 37.20 km [LTTF], P = 0.03). The frequency of clinic visits was statistically significantly different between adherence statuses: the adherent group had the highest number of clinic visits as compared to the NA and LTTF groups. Furthermore, the NA group had a higher average number of prescribed ocular medications than the other two groups (P = 0.03). Adherent patients had more frequent encounters with elevated IOP than the NA and LTTF groups (P = 0.02). There were no significant differences between the three adherence groups for the following parameters: requirement for surgery and EUA, glaucoma subtype (primary congenital glaucoma [PCG] vs. other glaucoma subtypes), and BCVA. BCVA was not a significant factor for determining the patient’s adherence status regardless of whether it was evaluated based on the vision in the best or worst eye (P > 0.05).

**Table 2:** Patient characteristics (n = 91) BCVA = best corrected visual acuity, LTTF = lost to tertiary follow-up, NA = non-adherent, OD = right eye, OS = left eye, PCG = primary congenital glaucoma

Variable	Category	Count	Percent
Nationality	Non-Saudi	21	27.9%
	Saudi	70	72.1%
Glaucoma type	PCG	83	84.5%
	Other	8	15.5%
BCVA (OD)	Poor	27	29.6%
	Fair	28	30.7%
	Good	36	39.7%
BCVA (OS)	Poor	32	35.1%
	Fair	26	28.5%
	Good	33	36.4%
Adherence status	Adherent	52	57.1%
	NA	20	22%
	LTTF	19	20.9%

**Table 3:** Sociodemographics of patients (n = 91) IOP = intraocular pressure, Min = minimum, Max = maximum

Variables	N	Min.	Max.	Mean±standard deviation
Age (years)	91	1	18	6.20± 4.42
Frequency of encounters	91	0	3	0.23± 0.56
IOP > 21	91	0	17	2.15± 2.93
Mean number of ocular medications	91	0	5	1.52± 1.13
Distance from clinic (km)	91	4	1469	72.06± 182.56

**Table 4:** Single-variable analysis of potential risk factors for non-adherence *P values indicate significance if < 0.05. EUA = examination under anesthesia, IOP = intraocular pressure, LTTF = lost to tertiary follow-up, N = number, NA = non- adherent, VA = visual acuity

Patient characteristics	Adherent	NA	LTTF	P	
Male sex, No. (%)	29 (53.7)	8 (40)	12 (63)	0.31	
Age, years	4.88	3.81	4.65	0.29	
Saudi nationality, No. (%)	38 (73)	17 (85)	9 (47)	0.02*	
Frequency of encounters per month, No.	2	0.8	0.72	0.007*	
Surgery required, No. (%)	46 (90)	18 (90)	18 (90)	0.38	
EUA required, No. (%)	43 (91.8)	15 (76.2)	22 (88.5)	0.19	
Number of visits with IOP > 21 mm Hg	3.11	1.85	1.10	0.02*	
Mean number of ocular medications	1.98	2.33	1.57	0.03*	
Mean distance from the clinic (km)	40.16	23.31	37.20	0.03*	
Paediatric glaucoma subtype PCG, No. (%)	1 (2)	0 (0)	1 (3.7)	0.522	
Other subtypes, No. (%)	48 (98)	21 (100)	26 (96.3)		
Vision in the right eye Good VA, No. (%)	22 (45.8)	8 (30.7)	6 (35.3)	0.65	
Fair VA, No. (%)	14 (29.1)	9 (34.6)	5 (29.4)		
Poor VA, No. (%)	12 (25.1)	9 (34.7)	6 (35.3)		
Vision in the left eye Good VA, No. (%)	18 (36.3)	10 (38.7)	5 (30.4)	0.68	
Fair VA, No. (%)	12 (25.4)	8 (32.2)	6 (34.7)		
Poor VA, No. (%)	19 (38.3)	7 (29.1)	6 (34.9)		

## Discussion

An estimated 60 million people worldwide are affected by PCG, which has caused blindness in 12 million people. The occurrence rate differs between societies and geographical borders, where it is 1 in 10,000-20,000 in Western countries [12]. PCG prevalence is higher in the Middle East, notably in Saudi Arabia, where consanguineous marriages are more common [3]. In 2014, approximately 251,736 live births were documented in Saudi Arabia [13]. Based on a PCG incidence of 1 in 2500-3000 live births, PCG might affect up to 100 infants in the Saudi population annually [14]. Consequently, the burden of people with glaucoma is markedly higher in Saudi Arabia, especially for children at the beginning of their lives. This indicates the necessity of early screening for early detection and a strictly managed follow-up visit policy. Glaucoma patients require constant treatment and follow-up care to retain their vision [15]. Many studies highlighted the importance of adhering to follow-up regimens, where increased glaucoma severity was associated with poor follow-up [16]. In the present study, we assessed the proportion of adherent, NA, and LTTF paediatric glaucoma patients and the factors associated with their adherence status. Of the 91 patients who were traced successfully, 39 patients (47%) failed to attend their scheduled appointment regularly or completely discontinued the assigned follow-ups. Given the extremely high percentage, the result confirmed the impressions of numerous clinicians that patient adherence to follow-up appointments is a significant issue in treating paediatric glaucoma. However, Mikolajczyk et al. [11] reported that only 3% of patients did not adhere to their follow-up appointments. On the contrary, the 22% NA rate in our study was markedly higher.

Our results demonstrated that adherence was consistently more strongly associated with the following variables: Saudi nationality, living farther away from the ophthalmology clinic, increased frequency of encounters per month, mean number of ocular medications, and high number of clinic visits with elevated IOP. No significant differences were detected between the three adherence groups for the following parameters: requirement for surgery and EUA, glaucoma subtype, or BCVA. It is well established that racial and ethnic disparities in health care reflect challenges with access to care and other factors brought on by varying socioeconomic circumstances [17-20]. The literature on racial disparities in health outcomes is substantial [17-20], and both hidden and explicit prejudices in our healthcare system are emerging as factors, which is relevant given that non-Saudi nationality is a key risk factor for poor adherence in our study. It will require investigation to identify the precise causes of racial disparities among our patients in our health care system and examine elements of the clinical experience that might be responsible for such inequalities to allow for the creation of solutions that specifically target those factors.

Although national and international data on adherence to paediatric glaucoma appointments are scarce, Mikolajczyk et al. reported that 54% of paediatric glaucoma patients were adherent to clinic visits [11]. Our main finding was that only 57% of the participants were adherent to follow-ups, which was similar to a previously reported percentage [11]. The equivalent setting (tertiary healthcare centre, retrospective record review design) could explain the similarity between both studies regardless of the entirely different populations (demography, socioeconomics, languages, further dimensions). Studies involving adults [21,22] highlighted compliance as low as 39% for recommended glaucoma appointments. Patients provided differing reasons for missing such appointments, such as forgetting the scheduled appointment [22], lack of transportation [22,23], or low economic status [22,24]. Other reasons for follow-up non-adherence in adult glaucoma patients included end-stage glaucoma and poorer visual acuity [24], limited literacy level [22,23], a lack of understanding of glaucoma and the significance of regular eye care [22], and black or Hispanic ethnicity [23,25]. Furthermore, glaucoma care costs are important in patients’ compliance with follow-up visits [26]. Moreover, the NA patients tended to live closer to the hospital than the adherent and LTTF patients. This finding was inconsistent with that of Mikolajczyk et al., who reported that NA patients lived farther away from the clinic. This might be attributed to the fact that patients living in rural areas farther away from the hospital might lack advanced healthcare services in their area and therefore have a compelling need to adhere to their appointments at tertiary care centres.

Adherence was also associated with increased visits with higher IOP. This could have been due to the poor outcomes the patients experienced and greater number of adverse events. It is essential to remember that the association between follow-up adherence and a specific parameter does not automatically indicate causality in either direction. Although follow-up adherence among adult glaucoma patients has been extensively investigated, ours is the first study to examine the factors associated with follow-up visit adherence in paediatric glaucoma patients in Saudi Arabia.

This study was limited by the retrospective design and the small sample size, primarily due to the improper coding of the paediatric glaucoma patients’ files within the hospital records. This resulted in insufficient retrieval of the total encountered patients. The lack of standardization of documented patient notes during clinic visits was another study limitation. Consequently, despite our attempts to obtain the optimum number of records, the percent adherence to follow-up of the cohort might have been overestimated. Exploring the additional factors that influenced the adherence status of paediatric glaucoma patients, such as socioeconomic status, educational level and caregivers’ awareness, was not feasible within our study scope, but further assessment is encouraged. More investigations involving hospitals with more glaucoma cases are needed. Interventional studies with a prospective cohort design are essential to increase adherence and reduce bias. For prompt diagnosis and treatment, more effective screening techniques may be useful [27]. Devastating visual results might result from delays in presentation, diagnosis, and management. The disease's underrepresentation among caregivers and professionals is the main cause of the delayed manifestation. Healthcare policies must conduct educational initiatives to raise caregivers' understanding of the importance of early intervention for PCG and to guarantee the early discovery of the condition, especially in developing countries or low-resource regions. Further studies should be conducted to aid recognition of the factors related to patient non-adherence and to propose solutions. This study targeted paediatric patients with glaucoma, and the results are likely to be generalizable to similar settings. Nevertheless, it is expected that access to care and support systems differ across populations, affecting the adherence status.

## Conclusions

This study highlighted the factors associated with strong adherence to follow-up appointments among paediatric glaucoma patients as compared to NA and LTTF patients. These factors included Saudi nationality, living farther away from the ophthalmology clinic, higher frequency of encounters per month, higher number of visits with elevated IOP, and the prescription of several ocular medications. In addition to maximizing doctor-patient communication to promote such compliance, clinicians should consider these risk variables to predict the likelihood of follow-up non-adherence.
